# Supplementary material for: Human attention during goal-directed reading comprehension relies on task optimization
Source: eLife. 2023 Nov 30;12:RP87197. doi: 10.7554/eLife.87197 (PMC10688971; doi:10.7554/eLife.87197)
Supplement: Supplementary file 1. — (a) p-values for the model prediction of word reading time. trans_rand: transformer-base models with randomized parameters; trans_pre: pre-trained transformer-based models; trans_fine: transformer-based models fine-tuned on the goal-directed reading task. (b) p-values for the prediction of word reading time using text or task-related features. (c) Linear mixed effects modeling of human word reading time. The question type is coded as 0 (local question) or 1 (global question), and other factors are continuous regressors. Given the substantial number of attention weights in BERT (i.e., 144), we present the 1st quartile and 3rd quartile values for b, SE, and t and report the ratio of attention weights that reach significant level. b: regression coefficient; SE: standard error of regression coefficient. (d) p-values for the prediction of early and late eye-tracking measures using text or task-related features. GD: gaze duration; CR: counts of rereading. (e) p-values for the prediction of word reading time for all four experiments. trans_pre: pre-trained transformer-based models; trans_fine: transformer-based models fine-tuned on the goal-directed reading task. (f) p-values for the comparisons between experiments. trans_pre: pre-trained transformer-based models; trans_fine: transformer-based models fine-tuned on the goal-directed reading task. (g) Hyperparameters for DNN fine-tuning. We adapted these hyperparameters from references (Lan et al., 2020; Liu et al., 2019; Zhu et al., 2015; Wolf et al., 2020). [file elife-87197-supp1.docx]

**Supplementary file 1a**

|  | | Cause | Fact | Inference | Theme | Title | Purpose |
| --- | --- | --- | --- | --- | --- | --- | --- |
| SAR | | 0.002 | 0.002 | 0.002 | 0.002 | 0.002 | 0.002 |
| trans_rand | | 0.002 | 0.002 | 0.002 | 0.002 | 0.002 | 0.002 |
| trans_pre | 0.002 | | 0.002 | 0.002 | 0.002 | 0.002 | 0.002 |
| trans_fine | | 0.002 | 0.002 | 0.002 | 0.002 | 0.002 | 0.002 |
| SAR vs. trans_rand | | 7×10^-5^ | 7×10^-5^ | 7×10^-5^ | 7×10^-5^ | 7×10^-5^ | 7×10^-5^ |
| SAR vs. trans_pre | | 7×10^-5^ | 7×10^-5^ | 7×10^-5^ | 7×10^-5^ | 7×10^-5^ | 7×10^-5^ |
| SAR vs. trans_fine | | 7×10^-5^ | 7×10^-5^ | 7×10^-5^ | 7×10^-5^ | 7×10^-5^ | 7×10^-5^ |
| trans_rand vs. trans_pre | | 7×10^-5^ | 7×10^-5^ | 7×10^-5^ | 7×10^-5^ | 7×10^-5^ | 7×10^-5^ |
| trans_rand vs. trans_fine | | 7×10^-5^ | 7×10^-5^ | 7×10^-5^ | 7×10^-5^ | 7×10^-5^ | 7×10^-5^ |
| trans_pre vs. trans_fine | | 7×10^-5^ | 7×10^-5^ | 9×10^-4^ | 0.351 | 0.468 | 0.278 |

**Supplementary file 1b**

|  | Cause | Fact | Inference | Theme | Title | Purpose |
| --- | --- | --- | --- | --- | --- | --- |
| layout | 0.002 | 0.002 | 0.002 | 0.002 | 0.002 | 0.002 |
| word | 0.002 | 0.002 | 0.002 | 0.002 | 0.002 | 0.002 |
| relevance | 0.002 | 0.002 | 0.002 | 0.002 | 0.002 | 0.002 |

**Supplementary file 1c**

|  | *b* | *SE* | *t* | *p* |
| --- | --- | --- | --- | --- |
| **Baseline** |  |  |  |  |
| (Intercept) | 67.60 | 2.91 | 23.26 | < 0.001 |
| QuestionType | 61.68 | 2.50 | 24.67 | < 0.001 |
| LeftMostPixel | -66.94 | 0.53 | -126.15 | < 0.001 |
| ParagraphNumber | 5.39 | 0.18 | 30.44 | < 0.001 |
| LineNumberInParagraph | -1.89 | 0.12 | -15.95 | < 0.001 |
| LineNumberInPassage | -5.96 | 0.07 | -89.35 | < 0.001 |
| WordLength | 16.55 | 0.08 | 211.84 | < 0.001 |
| LogWordFreq | -4.39 | 0.16 | -27.43 | < 0.001 |
| Surprisal | 2.19 | 0.08 | 26.72 | < 0.001 |
| QuestionRelevance | 336.87 | 1.52 | 221.54 | < 0.001 |
| QuestionType:LeftMostPixel | -38.59 | 0.88 | -44.05 | < 0.001 |
| QuestionType:ParagraphNumber | -12.05 | 0.32 | -38.04 | < 0.001 |
| QuestionType:LineNumberInParagraph | -15.12 | 0.20 | -76.80 | < 0.001 |
| QuestionType:LineNumberInPassage | 2.65 | 0.11 | 23.14 | < 0.001 |
| QuestionType:WordLength | -0.57 | 0.13 | -4.36 | < 0.001 |
| QuestionType:LogWordFreq | 0.52 | 0.27 | 1.96 | 0.05 |
| QuestionType:Surprisal | 0.46 | 0.14 | 3.34 | < 0.001 |
| QuestionType:QuestionRelevance | -93.03 | 2.72 | -34.258 | < 0.001 |
| **With attention of SAR** |  |  |  |  |
| SAR Attention | 2.12 | 0.11 | 19.41 | < 0.001 |
| **With attention of randomized BERT** |  |  |  |  |
| Randomized BERT attention | -0.57 ~0.61 | 0.2~ 0.22 | -2.7~ 2.75 | 64%<0.05  44%<0.001 |
| **With attention of pre-trained BERT** |  |  |  |  |
| Pre-trained BERT attention | -0.77 ~0.89 | 0.14~ 0.16 | -4.6~ 5.71 | 80%<0.05  67%<0.001 |
| **With attention of fine-tuned BERT** |  |  |  |  |
| Fine-tuned BERT attention | -0.72 ~1.01 | 0.14~ 0.16 | -4.9~ 6.81 | 86%<0.05  70%<0.001 |

**Supplementary file 1d**

|  |  | **local questions** | | |  | **Global questions** | | | |
| --- | --- | --- | --- | --- | --- | --- | --- | --- | --- |
|  |  | layout | word | relevance |  | layout | word | relevance |  |
| **Exp 1** | GD | 0.002 | 0.002 | 0.002 |  | 0.002 | 0.002 | 0.002 |  |
|  | CR | 0.002 | 0.002 | 0.002 |  | 0.002 | 0.002 | 0.002 |  |
|  | GD vs. CR | 4×10^-5^ | 4×10^-5^ | 4×10^-5^ |  | 6×10^-5^ | 8×10^-5^ | 6×10^-5^ |  |
| **Exp 2** | GD | 0.002 | 0.002 | 0.01 |  | 0.002 | 0.002 | 0.006 |  |
|  | CR | 0.002 | 0.002 | 0.002 |  | 0.002 | 0.002 | 0.002 |  |
|  | GD vs. CR | 0.704 | 1×10^-4^ | 1×10^-4^ |  | 1×10^-4^ | 0.333 | 0.333 |  |
| **Exp 3** | GD | 0.002 | 0.002 | 0.265 |  | 0.003 | 0.003 | 0.302 |  |
|  | CR | 0.002 | 0.002 | 0.002 |  | 0.003 | 0.003 | 0.315 |  |
|  | GD vs. CR | 6×10^-5^ | 6×10^-5^ | 8×10^-5^ |  | 0.656 | 1×10^-4^ | 0.724 |  |
| **Exp 4** | GD | 0.003 | 0.003 | 0.685 |  | 0.003 | 0.003 | 0.162 |  |
|  | CR | 0.003 | 0.003 | 0.405 |  | 0.003 | 0.003 | 0.096 |  |
|  | GD vs. CR | 6×10^-5^ | 6×10^-5^ | 0.280 |  | 1×10^-4^ | 7×10^-4^ | 0.552 |  |

**Supplementary file 1e**

|  | local questions | | | |  | global questions | | | |
| --- | --- | --- | --- | --- | --- | --- | --- | --- | --- |
|  | Ex 1 | Ex 2 | Ex 3 | Ex 4 |  | Ex 1 | Ex 2 | Ex 3 | Ex 4 |
| trans_pre | 0.003 | 0.003 | 0.003 | 0.003 |  | 0.004 | 0.004 | 0.004 | 0.004 |
| trans_fine | 0.003 | 0.003 | 0.003 | 0.003 |  | 0.004 | 0.004 | 0.004 | 0.004 |
| trans_fine - trans_pre | 0.003 | 0.003 | 0.041 | 0.814 |  | 0.9 | 0.9 | 0.829 | 0.714 |
| layout | 0.002 | 0.002 | 0.002 | 0.002 |  | 0.003 | 0.003 | 0.003 | 0.003 |
| word | 0.002 | 0.002 | 0.002 | 0.002 |  | 0.003 | 0.003 | 0.003 | 0.003 |
| relevance | 0.002 | 0.002 | 0.002 | 0.228 |  | 0.003 | 0.003 | 0.003 | 0.003 |

**Supplementary file 1f**

|  | local questions | | | |  | global questions | | | |
| --- | --- | --- | --- | --- | --- | --- | --- | --- | --- |
|  | Ex 1 vs Ex 2 | Ex 2 vs Ex 3 | Ex 2 vs Ex 4 | Ex 3 vs Ex 4 |  | Ex 1 vs Ex 2 | Ex 2 vs Ex 3 | Ex 2 vs Ex 4 | Ex 3 vs Ex 4 |
| trans_pre | 0.001 | 0.460 | 1×10^-4^ | 1×10^-4^ |  | 0.155 | 0.055 | 1×10^-4^ | 1×10^-4^ |
| trans_fine | 0.2 | 0.953 | 1×10^-4^ | 1×10^-4^ |  | 0.02 | 0.114 | 1×10^-4^ | 1×10^-4^ |
| trans_fine - trans_pre | 1×10^-4^ | 0.032 | 1×10^-4^ | 1×10^-4^ |  | 0.04 | 0.305 | 0.12 | 0.622 |
| layout | 1×10^-4^ | 0.014 | 1×10^-4^ | 1×10^-4^ |  | 0.021 | 1×10^-4^ | 0.307 | 1×10^-4^ |
| word | 0.001 | 0.001 | 1×10^-4^ | 0.001 |  | 0.003 | 1×10^-4^ | 1×10^-4^ | 0.003 |
| relevance | 1×10^-4^ | 0.002 | 1×10^-4^ | 1×10^-4^ |  | 1×10^-4^ | 1×10^-4^ | 1×10^-4^ | 0.496 |

**Supplementary file 1g**

| models | learning  rate | fine-tuning  steps | fine-tuning  batch size | warmup steps | weight  decay |
| --- | --- | --- | --- | --- | --- |
| BERT | 1×10^-5^ | 27455 | 16 | 0 | 0 |
| ALBERT | 2×10^-5^ | 12000 | 32 | 1000 | 0 |
| RoBERTa | 1×10^-5^ | 21964 | 16 | 1200 | 0.1 |
